# Chiral Hierarchies at the Nanoscale Revealed by Three-Dimensional Scanning Electron Diffraction

**DOI:** 10.1021/acsnano.5c12291

**Published:** 2025-09-30

**Authors:** Mathias Nero, Mads Carlsen, Marianne Liebi, Tom Willhammar

**Affiliations:** † Department of Chemistry, 7675 Stockholm University, Stockholm SE-106 91, Sweden; ‡ Photon Science Division, 11248 Paul Scherrer Institute(PSI), Villigen 5232, Switzerland; § Institute of Materials, Ecole Polytechnique Fédérale de Lausanne (EPFL), Lausanne 1015, Switzerland

**Keywords:** scanning electron diffraction, chirality, cellulose, 3D reconstruction, electron diffraction

## Abstract

Natural biocomposites such as wood and plant cell walls exhibit prominent mechanical properties largely attributed to the nanoscale organization of fibrous components, such as cellulose, which often adopt chiral arrangements. However, resolving the three-dimensional (3D) arrangement of these structures at the nanoscale remains a significant challenge, particularly in beam-sensitive materials. This study introduces a method for 3D reconstruction of orientation based on scanning electron diffraction (SED), enabling the quantitative mapping of chiral supramolecular organization with sub-100 nm spatial resolution. By acquiring low-dose SED data at multiple tilt angles and applying a symmetry-based reconstruction algorithm, we resolved the 3D orientation of cellulose fibrils in native oat husk and birch wood. Our results reveal a multilayered cell wall architecture with alternating helical handedness, providing precise measurements of 3D fibril orientation. This method reveals complex hierarchical structures at the nanoscale, enabling rapid data acquisition and analysis using widely available instrumentation. The ability to resolve such chiral organization provides insights into material properties as well as opportunities for designing bioinspired materials with tunable mechanical and functional properties that extend far beyond natural biocomposite materials.

## Graphical abstract

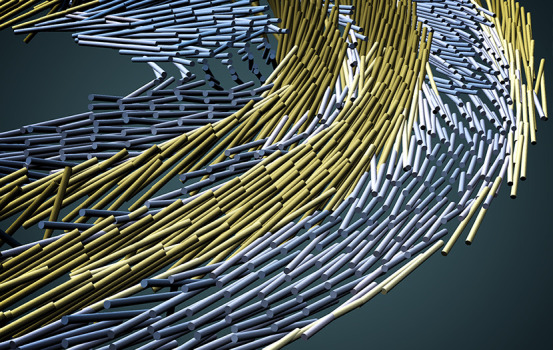

Nature has created a considerable amount of substances and hybrid materials built from organic compounds possessing chiral structures and assemblies, e.g., polysaccharides, amino acids, proteins, and DNA. This chirality typically originates at the molecular scale and can be transferred from molecular to macromolecular and supramolecular hierarchies. Structural organization is a defining factor for the properties of supramolecular and composite materials, impacting a range of attributes, from the mechanical strength of biobased hybrid materials^1^ to heat transport in nanocomposites.^2^ Lightweight yet strong biopolymeric materials often exhibit structural organization and anisotropy, enhancing their mechanical and functional properties across multiple length scales.^3,4^ For instance, plant cell walls derive strength and flexibility from the ordered arrangement of cellulose fibrils integrated with lignin and hemicellulose.^5^

Studies of hierarchical molecular organization pose high demands on structural characterization across length scales. A method capable of distinguishing chiral organization with nanoscale resolution will offer crucial insights for designing supramolecular and biomimetic materials with enhanced mechanical and functional properties.

Several methods have been used to image cell wall structures in two and three dimensions. Transmission Electron Microscopy (TEM)^6^ and Atomic Force Microscopy (AFM)^7^ have been used to reveal the two-dimensional organization of nanofibrillar cellulose in wood cell walls. X-ray phase-contrast nanotomography^8^ and electron tomography^9^ have been used to obtain 3D image reconstructions with nanoscale resolution, however, lacking quantitative information on the fibril orientations. To fully comprehend the molecular assemblies, their chirality must also be understood. Using polarized light, the dichroism or birefringence can be exploited to study chirality at the micrometer scale.^10^

Scattering methods, based on X-rays^11^ and neutrons,^12^ can effectively provide quantitative 3D information on structural arrangements, enabling the retrieval of both in-plane and out-of-plane orientation from thin samples, however, with a spatial resolution limited to the micrometer range. 3D scanning SAXS was developed to obtain 3D organization of fibrils in the call wall ultrastructure.^13^ Furthermore, SAXS and WAXS tensor tomography enable mapping of 3D objects, for instance, the 3D organization of mineralized collagen fibrils in bone.^14,15^ Texture tomography was demonstrated with a resolution of 500 nm, revealing the helical arrangement of a silica biomorph.^16^ Nevertheless, the spatial resolution of these methods is constrained by the size of the beam, and experiments can be relatively time-consuming due to the slow mechanical scanning of the sample.

Electron diffraction takes advantage of the strong interaction between electrons and matter, enabling scattering from nanometer-sized volumes. In recent years, four-dimensional scanning electron diffraction (4D-STEM) methods have made it possible to obtain spatially resolved electron diffraction data.^17^ Scanning electron diffraction (SED) is a part of 4D-STEM, utilizing a quasi-parallel beam to scan the sample in a raster pattern. By capturing diffraction patterns at each beam position, this technique enables detailed mapping of local crystallinity with nanometer spatial resolution.^18^ This process generates a four-dimensional data set, where each spatial coordinate corresponds to a diffraction pattern in reciprocal space, enabling high-resolution mapping of crystalline structures and providing insights into the organization of crystalline nanoparticles. Postprocessing analysis of SED data generates quantitative maps of crystal orientation, revealing nanoscale structural details with exceptional precision. SED has been utilized to study molecular packing within twisting chiral cellulose nanofibers^19^ as well as kinking cellulose nanocrystals,^20^ investigate the in-plane molecular arrangements in organic semiconductors^21^ and polymers,^22^ and examine individual dislocations in organic molecular crystals.^23^ SED has also been employed to assess the in-plane orientation of cellulose fibrils within a biobased composite derived from birch wood.^24^ Three-dimensional reconstructions of scanning electron diffraction data have been shown to map the morphology and orientation of highly crystalline materials.^25,26^ These results prompted further research and led to the exploration of three-dimensional mapping of soft semicrystalline anisotropic composite materials.

In this study, we present a novel method for 3D reconstruction of SED data, three-dimensional scanning electron diffraction (3D-SED), to examine the nanoscale chiral organization of cellulose in biocomposites. This approach advances the booming research field of SED and 4D-STEM by providing new structural insights. Our method combines low-dose SED and a geometric model of the electron scattering, providing high spatial resolution through the nanosized electron beam. This allows for detailed characterization of the hierarchical organization of weakly scattering anisotropic structures. By assuming axial symmetry of the nanocrystalline texture, we minimize the necessary tilt angles, making it particularly suitable for beam-sensitive specimens.

Herein, the method reveals the chiral arrangements of crystalline cellulose fibrils in plant cell walls with a spatial resolution below 100 nm. Notably, we uncover the nanoscale layer-by-layer reversal of chiral handedness within the cell walls of oat husk. By collecting SED data from several tilt angles, we are able to produce high-resolution 3D orientation maps across a 20 × 20 μm field of view, emphasizing the potential of 3D-SED as a valuable tool for investigating molecular organization with an unparalleled spatial resolution on structural organization.

## Results and Discussion

3D-SED was applied to two types of native composite biomaterials: oat husk and birch wood. Our analysis revealed the anisotropic arrangement and chiral organization of cellulose fibrils within these materials, demonstrating the feasibility of the method to resolve complex structural organization at the nanoscale.

3D-SED data were collected by scanning areas ranging from approximately 5 × 5 μm to 20 × 20 μm using a transmission electron microscope with a near-parallel electron beam (convergence semiangle of 0.09 mrad) roughly 8 nm in diameter (see Figure S6), with step sizes varying from 40 to 200 nm to provide both an overview and more detailed characterization. Each area was scanned sequentially at several distinct tilt angles around a single tilt axis (Figure 1a), yielding 10,000 to 20,000 diffraction patterns from each tilt angle.

**Figure 1:**
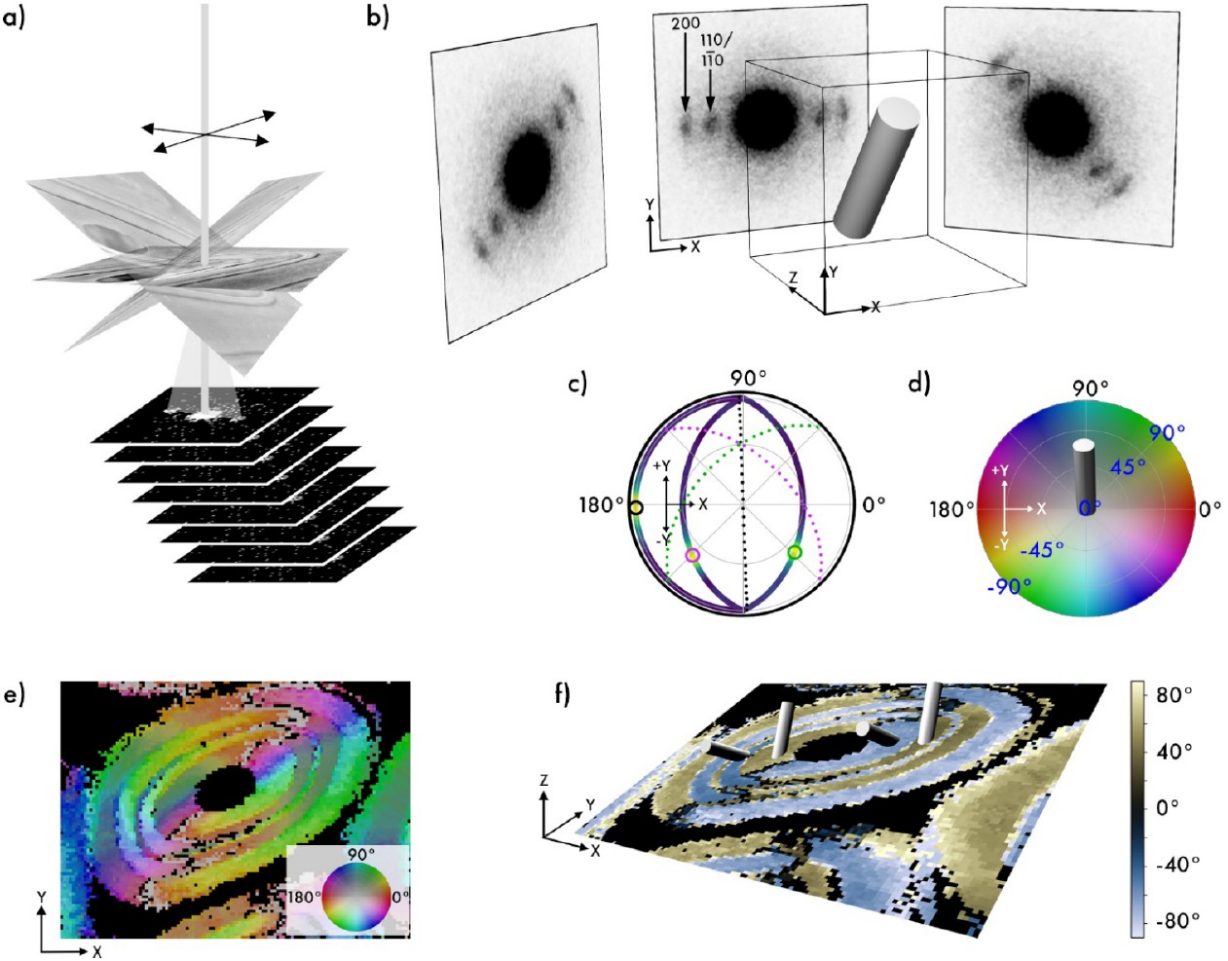
Three-dimensional scanning electron diffraction. (a) 3D-SED data were collected by scanning the sample area with a nanometer-sized, near-parallel electron beam in a raster pattern at several tilt angles. (b) Schematic illustrating an example pixel containing a fibril and how tilting the sample affects the azimuthal angle of the diffraction patterns, with the three most prominent reflections indexed. The azimuthal angle for each diffraction pattern was determined from the average intensities of 360 virtual detectors arranged in a ring around the direct beam at a radius corresponding to the g-vector of the (200) reflection. (c) Pole figure for the same example pixel, displaying azimuthal scattering curves from three tilt projections transformed into a common coordinate system and plotted with the viridis color scheme. Circles mark peak positions, dotted lines indicate orientation constraints, and the best-fit 3D fibril orientation from iterative projection averaging. (d) Color wheel for visualizing reconstructed 3D orientations, with a cylinder marking the best-fit orientation of the example pixel in (b) and (c). (e) Orientation map of the three-dimensional fibril directions following the color wheel in (d), with black denoting regions lacking significant signal. (f) Cylinder plot of the same data shown in (e), where background colors indicate positive or negative Y-components of the reconstructed vector, with the out-of-plane angle indicated by the color bar on the right. The innermost layer forms a left-handed helix, while the next layer forms a right-handed helix.

One of the primary challenges in applying electron microscopy to organic or other beam-sensitive materials is radiation damage, resulting in a gradual loss of crystallinity. To mitigate this, a low beam current of 2 pA and an exposure time of 5 ms were used, resulting in an electron dose of approximately 10 e^–^/Å^2^. The use of undersampling by using step sizes larger than the beam size further reduced potential radiation damage. Under these conditions, the diffraction patterns will reveal features at the length scale relevant for detecting molecular-scale packing, 1–10 Å. For each collected diffraction pattern, the azimuthal angle of Bragg diffraction was analyzed and quantified using 360 virtual detectors arranged around the direct beam to generate an intensity profile. In this example, the diffraction patterns were indexed according to the cellulose Iβ crystal structure (Figure 1b), which describes the arrangement of glucan chains within the cellulose fibrils.^27^

To enable orientation-mapping from few tilt angles, we assume rotational symmetry around one axis of the nanocrystalline texture in the probed volume, fixing the reciprocal lattice (001) direction to a symmetry axis â. Here, the orientation analysis is based on the most intense diffraction peak 200. According to the Iβ crystal structure of cellulose, this reflection describes the intermolecular spacing between glucan chains, and the diffraction vector occurs normal to the extended dimension of the cellulose fibril. The azimuthal angle with the maximum intensity of the Bragg peak, obtained from each diffraction pattern, Figure 1b, then corresponds to a q-vector normal to â, in principle fixing the in-plane angle of â.

To recover the out-of-plane angle, we repeat the measurement with a tilted sample (Figure 1a). By aligning and stretching the tilted images and by rotating the measured q-vectors to a common zero-tilt coordinate system (Figure 1c), we obtain a number of orientations *q*_*i*_ normal to â. The problem is then to find an axis â normal to all *q*_*i*_. For two measurements, this is exactly solved by the cross-product $â\left|\right|\frac{q_{1}\times q_{2}}{\left|q_{1}\times q_{2}\right|}$ but for more than two measurements, there will in general not be a single orientation normal to all *q*_*i*_ and we must determine some best-fit orientation that is approximately normal to the set of measurements. This can be achieved by a range of different approaches. Here, we use an iterative averaged projections approach, for more details see the Methods section. To effectively visualize the reconstructed three-dimensional data, we are using two plotting methods. Because the sign of the symmetry axis, â, which denotes the extended axis of the fibril, is arbitrary, there are always two equivalent unit vectors describing the orientation, which leads to complications in visualizing the orientation. Any RGB color-coding of orientation will either have multiple distinct orientations with the same color or discontinuities.^28^ The first method (Figure 1e) utilizes hue to represent the in-plane orientation, while saturation indicates the out-of-plane angle, with 0° defined as perpendicular to the xy-plane and 90° as parallel to the xy-plane. Furthermore, light and dark colors differentiate between the positive and negative y-components (after restricting directions to the positive-z half-unit-sphere) based on the color wheel in Figure 1d. The second visualization method is a plot where cylinders depict the 3D vector of the reconstructed fibril orientation (Figure 1f). To emphasize the two distinct chiralities in the cylinder plot, the background of Figure 1f depicts the orientations of cellulose fibrils using the out-of-plane component, with yellow representing those oriented with a positive y-component and blue indicating a negative y-component (see Figure S1). To show only structurally relevant regions, a threshold was applied to exclude pixels lacking defined Bragg diffraction, rendering these areas black. Due to the single-tilt-axis geometry, fibril orientations with a zero y-component cannot be unambiguously determined, leading to unmapped pixels along specific directions.

### Cell Wall of Oat Husk

The oat husk, which constitutes the outermost protective layer of the grain, is a multilayered structure composed of two floral bracts: the lemma and the palea.^29^ Details about the orientation of cellulose within the husk cell walls are still limited. However, studies have shown that the cell walls of epidermal cells can exhibit a crossed-polylamellated or helicoidal structure.^30^ An Annular Dark-Field Scanning Transmission Electron Microscopy (ADF-STEM) image, Figure 2a, shows a transversely sectioned oat husk cell from the lemma with a slightly elliptical cross-sectional shape composed of distinct cell wall layers ranging in thickness from a few hundred nanometers to several micrometers. Initial 3D-SED data were obtained from this area with a step size of 100 nm, revealing its multilayered structure and fiber organization.

**Figure 2:**
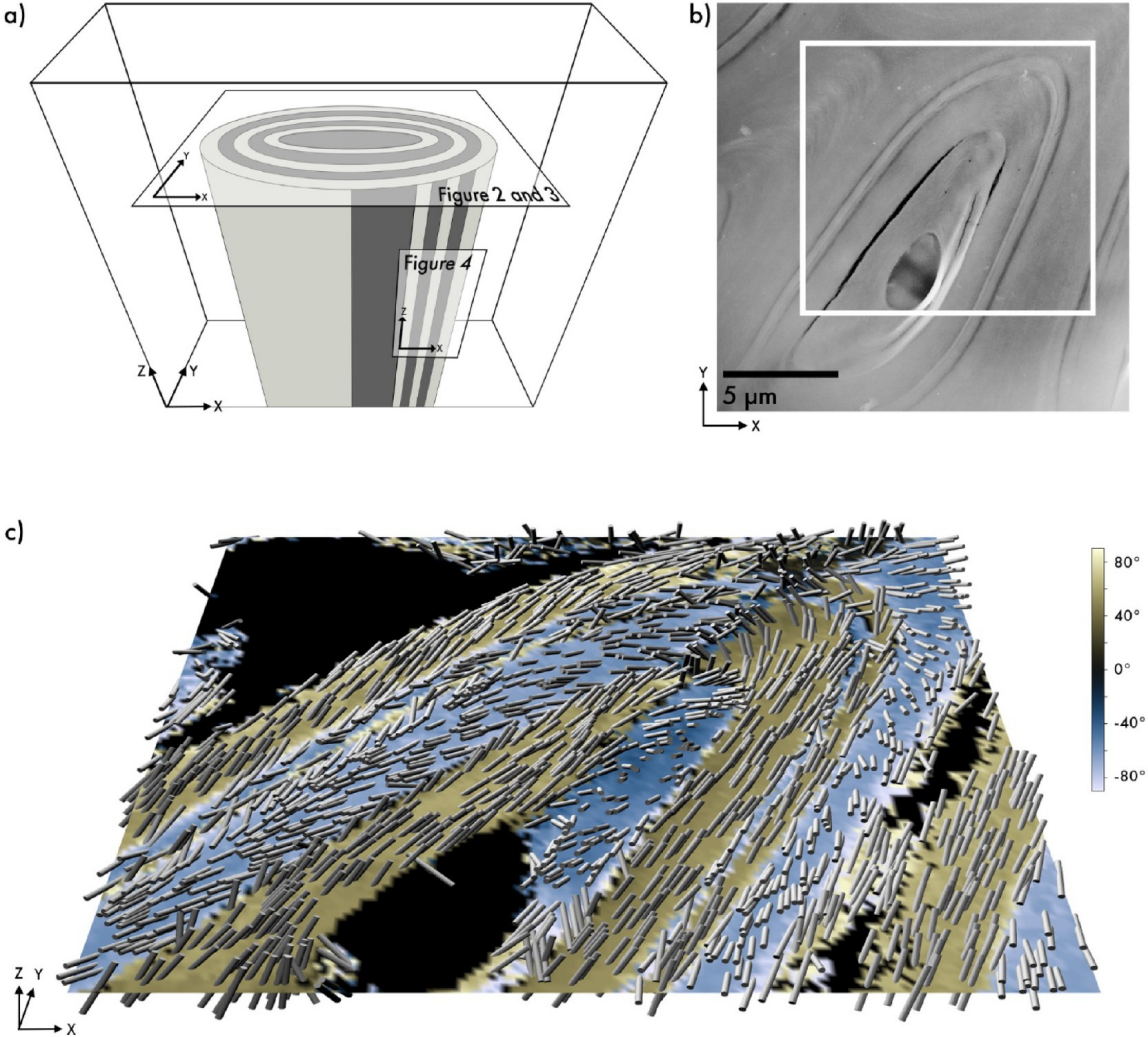
3D-SED data reconstruction of a transversely sectioned oat husk cell. (a) Schematic showing longitudinal and tangential sectioning of an oat husk cell. (b) ADF-STEM image of an almost complete oat husk cell, with the scanned area highlighted. (c) 3D cylinder plot illustrating the opposing chiral arrangement of cellulose fibrils across different layers, following the cell shape tangentially. Out-of-plane orientation, with positive or negative Y-components of the reconstructed vector, is shown in yellow and blue, with the corresponding out-of-plane angle indicated by the color bar on the right.

The high-resolution color plot in Figure 2b provides quantitative three-dimensional insights into the fibril organization, showing fibrils oriented tangentially along the cell circumference. The 3D reconstruction reveals an innermost layer that exhibits a left-handed helical organization, indicated by dark green regions on the left side of the lumen and light green on the right side. Numerical averages of the out-of-plane angles in each layer shown in Figure 3 are presented in Figure S2. The average helix angle, defined as the angle between the helical path and the extended axis of the cell, is approximately 33° (see Table S1). The pitch, which is the distance along the extended axis of the cell for a complete loop, can be calculated based on the helix angle and circumference (see Figure S2). For example, the inner layer, with a circumference of 31 μm, has a pitch of 25 μm. Tabulated pitch values for all layers depicted in Figure 1 can be found in Table S1. The 3D reconstruction illustrated in the cylinder plot of Figure 2c reveals the orientation of cellulose fibrils within each layer. This demonstrates a radially alternating transition between left- and right-handed helical organizations across layers, as highlighted by the varying background colors.

**Figure 3:**
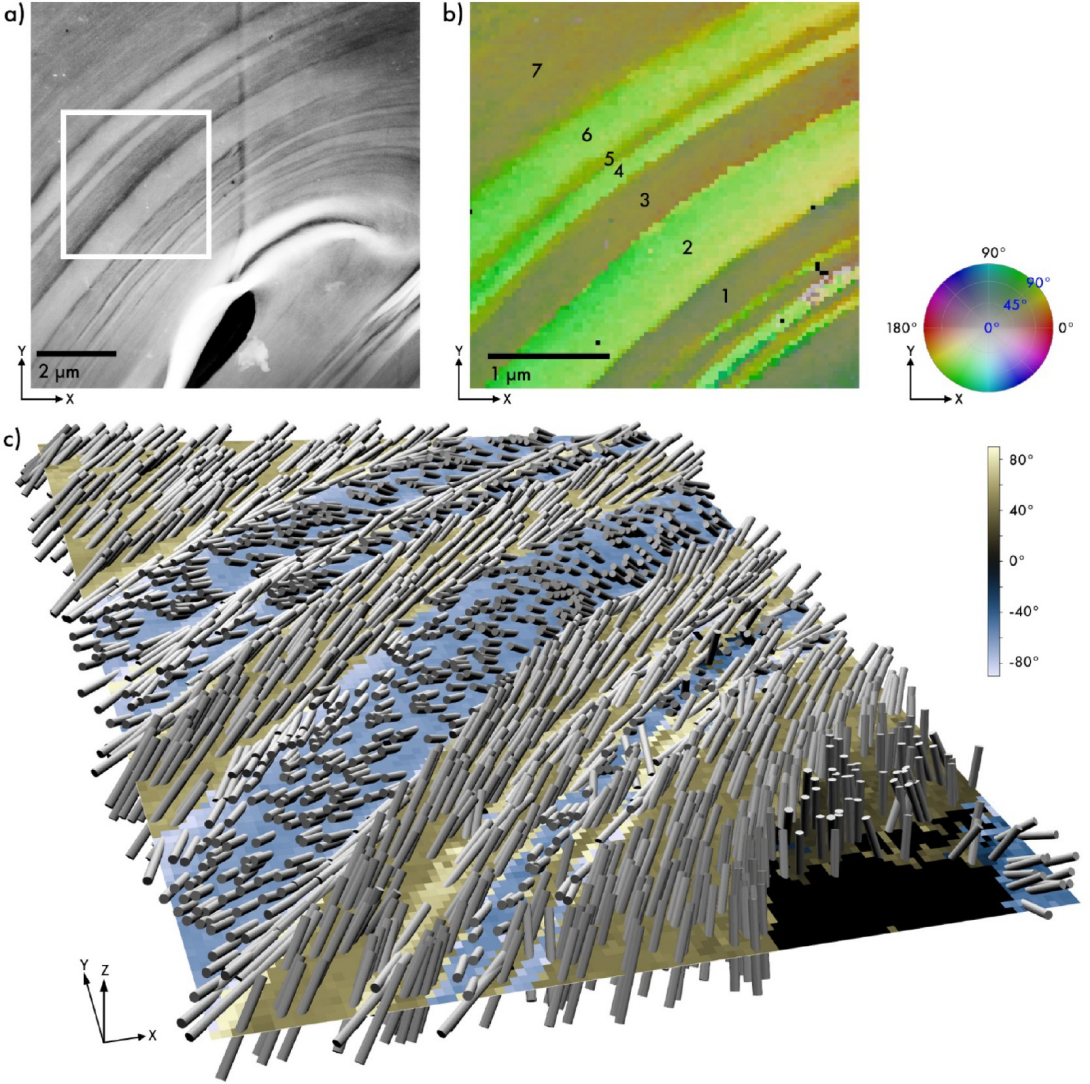
3D-SED reconstruction demonstrating the nanometer-scale spatial resolution of this method from a transversely sectioned oat husk cell. (a) ADF-STEM image showing approximately one-quarter of an oat husk cell, highlighting its multilayered structure. The white square marks the 3D-SED scanning area. (b) Color plot of the 3D reconstruction, collected with a 40 nm step size, showing the tangential in-plane orientation of nanofibers (mainly green in this region) and alternating out-of-plane orientations (dark and light green) according to the color wheel. With the lumen located in the lower right corner, dark green layers correspond to a left-handed arrangement, and vice versa. (c) 3D cylinder plot showing the layer-by-layer alternation in fibril orientation chirality, with fibrils aligned tangentially to the cell wall. Positive and negative Y-components of the reconstructed vector, representing the out-of-plane orientation, are shown in yellow and blue, with the associated angle indicated by the color bar on the right.

The multilayered cell wall structure with alternating helical handedness can be generally observed in transversely sectioned oat husk. A second data set, collected using a finer step size of 40 nm, was reconstructed (see Figure 3) with the lumen region located in the lower-right corner. The 3D reconstruction effectively highlights the alternating chiral orientation of cellulose fibrils across multiple layers, as shown in Figure 3b. The higher spatial resolution allows for the reconstruction of cell wall layers as thin as <100 nm, revealing the alternating handedness of consecutive layers at the nanoscale (Figure 3b).

Table 1 presents fibril orientation within each cell wall layer based on the reconstructed orientations, where the alternating sign of the out-of-plane angle illustrates the shifts in helical handedness. As the lumen is located at the bottom right, negative out-of-plane angles correspond to a right-handed helix. It can be observed that both the right-handed and left-handed layers exhibit consistent orientations, although accurately quantifying the thinner layer 5 proves to be challenging. Left-handed layers have an average in-plane orientation of 29(3)° and out-of-plane orientation of 60(4)°, and the right-handed layers have an in-plane orientation of 45(2)° and −63(2)°, respectively. To assess the uncertainty of the reconstruction, additional reconstructions were performed based on sets of generated azimuth-directions with random noise, and the effect of this noise on the recovered fibril directions was examined. Under these assumptions, we found that standard deviations of around 3° align with the noise level of the measurements (see Figure S7).

**Table 1:** Fibril Orientation in the Cell Wall Layers of Transversely Sectioned Oat Husk, from Figure 3b

Nanofibril orientation		
	In-plane	Out-of-plane
Layer 1	33°	56°
Layer 2	47°	–61°
Layer 3	27°	60°
Layer 4	43°	–62°
Layer 5	36°	78°
Layer 6	45°	–65°
Layer 7	27°	63°

The orientation relationship between fibrils in adjacent cell wall layers can be calculated using the absolute value of the dot product. Where the average orientation relationship is 60(5)° between consecutive layers of opposite handedness in transversely sectioned oat husk, see Figure S3.

A 3D-SED data set from longitudinally sectioned oat husk cells with a spatial resolution of 40 nm was obtained and reconstructed. The ADF-STEM image in Figure 4a shows a side-view segment of an oat husk cell wall, featuring the lumen area at the bottom and the multilayered cell wall structure above. The middle lamella, separating adjacent cells, does not contain aligned cellulose and appears as a dark band.

**Figure 4:**
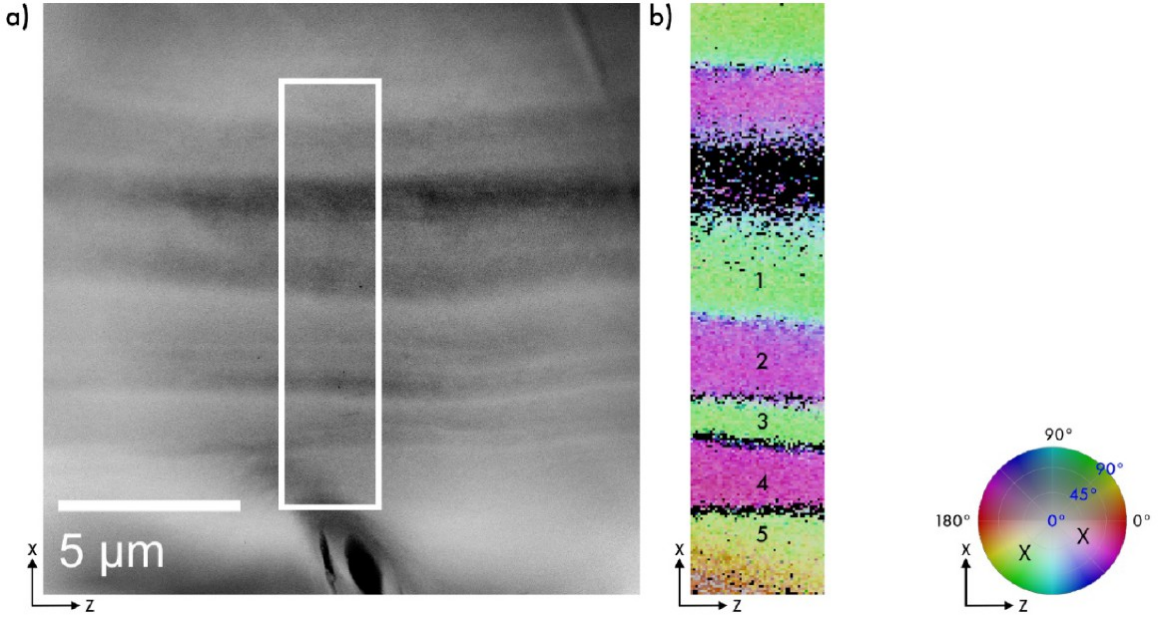
Three-dimensional reconstruction of a longitudinally sectioned oat husk cell. (a) ADF-STEM image showing a side view of an oat husk cell wall (as in the schematic in Figure 2a), with the lumen at the bottom and the multilayered cell wall structure above. (b) Color plot of the 3D reconstruction from data collected within the white rectangle in (a), obtained with a 40 nm step size and colored according to the color wheel, with the two predominant orientations approximately marked by X. In contrast to the transverse sectioning shown in Figures 2 and 3, this viewing direction reveals alternating chirality from changes in the in-plane orientation, while the out-of-plane orientation remains relatively stable (see Table 2).

The 3D reconstruction reveals fibril orientations consistent with those observed in transverse sections. Specifically, the in-plane fibril orientations in the longitudinal sections correspond to the out-of-plane angles measured from the transverse sections. The out-of-plane orientations exhibit the same sign for all layers, in this case, a negative y-component, which is consistent with the longitudinal sectioning of an elongated cell. The alternation of the chiral organization is reflected in the changes in the in-plane orientations. Minor variations (approximately ± 10°) observed in the out-of-plane orientation of fibrils align well with the range of in-plane variations documented in the transverse sections. Table 2 presents the fibril orientation layer-by-layer in this region of the cell. In this viewing direction, the average orientation relationship of fibrils between layers, as obtained using the absolute value of the dot product, is 77(5)° (see Figure S3b).

**Table 2:** Fibril Orientation in the Cell Wall Layers of Longitudinally Sectioned Oat Husk, from Figure 4b

Nanofibril orientation		
	In-plane	Out-of-plane
Layer 1	155°	–67°
Layer 2	54°	–51°
Layer 3	147°	–61°
Layer 4	58°	–54°

3D-SED data reveals the alternating chiral organization of cellulose fibrils across the multilayered cell walls of oat husks, potentially improving their biomechanical properties, at nanoscale resolution. The technique enables the study of chiral layers less than 100 nm in thickness. Quantitative analysis determined both the orientation of the fibrils and their relative alignment across layers. Collectively, these findings provide a comprehensive and representative view of the structural complexity of oat husks. To our knowledge, this is the first study revealing the chiral organization in oat husk cell walls at this level of detail.

### Cell Wall of Birch Wood

Wood cells are known to possess a helical organization of cellulose fibrils with varying orientations across the layers of the secondary cell wall, comprising a thin outer S1 layer, a thick middle S2 layer, and a thin inner S3 layer adjacent to the lumen.^31^ The organization of cellulose fibrils at the S1–S2 interface is disputed. Studies have revealed a radial orientation of cellulose fibrils; whether this configuration represents a crisscross^32^ or whorl-like^33^ arrangement of cellulose has remained unresolved.

Birch wood was sectioned transversely and longitudinally. Figure 5a presents a ADF-STEM image of transversely sectioned birch wood where two fiber cells are joined by the middle lamella, with lumen regions on each side (as shown in Figure 5c). The corresponding 3D-SED reconstruction (Figure 5b), collected with a 100 nm step size, demonstrates that fibrils in the thick S2 layer align close to the direction of cell elongation with a small deflection along the *y*-direction. Specifically, in the left cell, fibrils within this layer orient along a direction corresponding to a positive *y*-axis.

**Figure 5:**
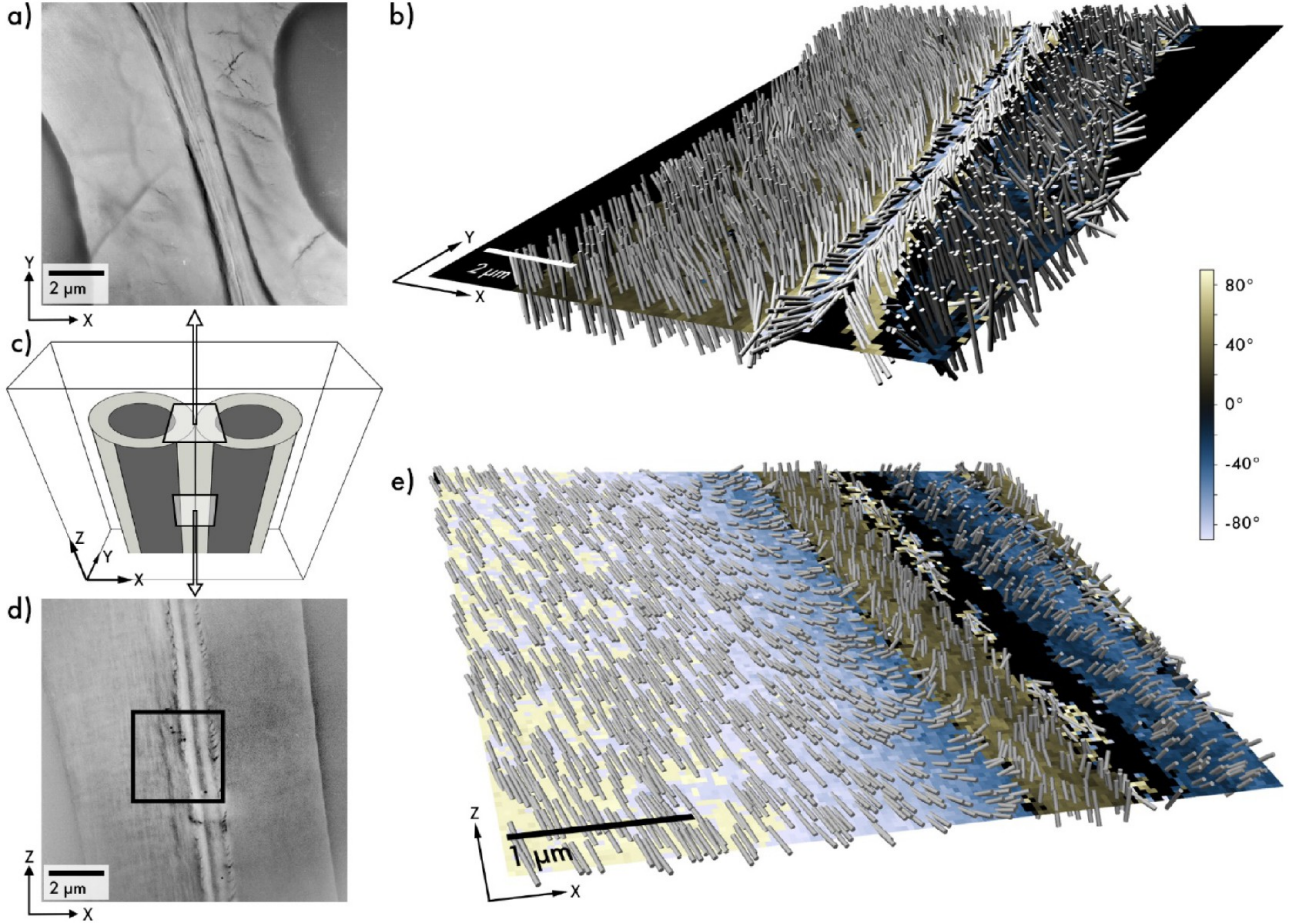
Three-dimensional scanning electron diffraction (3D-SED) reconstruction illustrating the chiral arrangement of cellulose fibrils in birch cell walls. (a) ADF-STEM image showing two transversely sectioned fiber cells joined by the middle lamella, with lumen regions visible on both sides. (b) 3D cylinder plot reconstructed from 3D-SED data covering the whole area shown in (a), collected with a 100 nm step size. In the thick inner S2 layer adjacent to the lumen, fibrils predominantly align along the cell elongation with an out-of-plane angle of approximately ± 26°. In the thin outer S1 layers, fibrils are oriented with an out-of-plane angle of approximately ± 73°. Based on out-of-plane orientation and lumen positioning, the fibrils exhibit right-handed helices in the S2 layers and left-handed helices in the S1 layers. (c) Schematic illustrating the sectioning orientation; the transverse and longitudinal data sets originate from different cells. (d) ADF-STEM image showing two longitudinally sectioned birch cells with lumen regions on both sides. (e) 3D reconstruction from the area marked in (d), collected with a 40 nm step size, showing cellulose fibril orientation with background colors indicating positive or negative Z-components of the reconstructed vector. Fibrils on the left side (in the S2 layer) align along the cell elongation in both in-plane and out-of-plane orientations. Near the outer layer, the fibrils rotate around the *X*-axis by approximately 120°, resulting in an out-of-plane orientation of 32° with a positive Z-component in the S1 layer to the left. The adjacent S1 layer in the neighboring cell displays an out-of-plane orientation of −33° with a negative Z-component (see Figure S4b). With the lumen on opposite sides, both outer layers exhibit a left-handed helical arrangement.

In contrast, in the cell to the right, fibrils exhibit alignment toward a negative *y*-axis direction in the S2 layer. This, and the lumen location, is consistent with a right-handed helical arrangement in both S2 layers with an out-of-plane angle of around 25(1)° (see Figure S4a). Adjacent to the middle lamella are two thinner layers of the cell wall. In these S1 layers, fibrils show an orientation closer to the sectioning plane. In the left cell, fibrils are directed toward the negative y-component, while in the cell to the right, fibrils in the thin S1 layer are directed toward the positive *y*-axis. In both cases, these S1 layers exhibit a left-handed helical orientation with an out-of-plane angle of 73(1)° (see Figure S4a). The orientation relationship of fibrils between the S1 and S2 layers is approximately 78° (see Figure S5a). In wood science, the out-of-plane angle in transversely sectioned wood is typically referred to as the microfibril angle (MFA).

3D-SED were obtained from a longitudinal section of birch wood fiber cells; see Figure 5d,e and the schematic in Figure 5c. The ADF-STEM image (Figure 5d) shows two cells separated by the middle lamella, with lumen regions visible on both sides. The SED data from this location were collected using a finer step size of 40 nm, providing higher spatial resolution. In the thick S2 layer (left side in Figure 5e), cellulose fibrils align parallel to the longitudinal direction of the elongated cell, as shown by an out-of-plane angle close to 90° (see Figure S4b). Approaching the thin S1 layer, the fibrils change from right- to a left-handed chiral arrangement around the lumen, resulting in a positive 32° out-of-plane angle in the S1 layer. A change in handedness has been suggested to relieve stress and potentially avoid collapse under negative pressure.^34^ The 3D-SED reconstructions show that the transition from S2 to S1 follows a smooth angular transition of approximately 120°, occurring without any spiraling or intersecting fibrils. Instead, the fibrils maintain a consistent left-handed trajectory throughout the transition (see Figure S5). It is known that cellulose nanocrystals extracted from wood form chiral nematic phases, which represent a transfer of chirality from the fundamental cellulose units to their hierarchical assembly.^35^ The left-handed transition between cell wall layers in the wood cell wall is consistent with the handedness of the helical organization in these chiral nematic phases. The smaller step size in this data set enables a quantitative characterization of the transition zone between layers, as illustrated in the graph in Figure S5c. The left-handed orientation of the thin S1 layer is also observed in the adjacent cell to the right. The consistent observations in both viewing directions confirm the chiral structural arrangement of cellulose in the birch fiber cell wall.

## Conclusion

This study introduces a novel method for three-dimensional reconstruction of scanning electron diffraction data, enabling the nanoscale analysis of chiral organization. The results demonstrate the effectiveness of this approach in mapping the anisotropic architecture of natural biopolymers such as cellulose in composite materials. By reconstructing data sets acquired at multiple tilt angles, we achieved high-resolution, quantitative characterization of fibril orientation.

Our findings highlight the complex chiral architecture of cellulose fibrils in native birch wood and oat husks. In the cell wall of oat husk, we quantitatively revealed alternating left- and right-handed helical arrangements of fibrils within successive layers, highlighting a complex yet highly ordered chiral architecture previously inaccessible with conventional techniques. In the cell wall of birch wood, 3D-SED discloses a continuous left-handed transition of the cellulose fibril orientation from the inner toward the outer cell wall.

These insights provide a deeper understanding of nature’s hierarchical design and offer a foundation for the development of bioinspired composite materials with tunable mechanical properties. With its high spatial resolution and efficient acquisition, 3D-SED emerges as a powerful and accessible complement to established techniques, offering new possibilities for structural characterization in materials science and sustainable engineering of biomimetic materials. The method extends far beyond the natural biocomposites and can be applied to engineered materials based on supramolecular organization, where the technique offers a pathway for enhanced understanding and engineering of advanced materials.

## Methods

### Materials

This study investigated two cellulose-containing native materials: birch wood (density of 620 kg/m^3^ from Glimakra of Sweden AB) and oat husk (provided from Lantmännen, Sweden, Oct 2023). Small specimens (1 × 1 × 10 mm) of each material underwent dehydration through a graded series of ethanol (50–100%) and were subsequently infiltrated with LR White resin. Resin polymerization was performed at 60*°*C for 24 h. Approximately 200 nm thick sections were prepared at room temperature using a Leica Ultracut UCT ultramicrotome fitted with a 35° diamond knife (Diatome). These sections were later transferred to carbon-coated copper grids (EMS-CF150-Cu-UL) for characterization.

### Data Acquisition

Data were acquired using a double aberration-corrected Thermo Fisher Themis-Z transmission electron microscope operating at 300 kV and equipped with a CheeTah M3 hybrid electron camera (512 × 512 pixels) from Amsterdam Scientific Instruments. ADF-STEM images and SED diffraction data were collected at a camera length of 670 mm. ADF-STEM imaging used a beam convergence angle of 3 mrad, a beam current of 30 pA, and an exposure time of 16 μs. 3D-SED data were obtained with a beam current of 2 pA, a convergence angle of 0.09 mrad, and a dwell time of 5 ms. Scanning of the beam was managed via the Gatan Microscopy Suite, while the CheeTah camera operated in continuous mode at 200 frames per second. 3D-SED data sets were recorded at three tilt angles: 0°, + 45°, and −45°, relative to the optical axis, with the tilt axis oriented along the vertical direction of the grid.

### Data Processing

SED data were processed using custom Python scripts. At low magnifications, a small but systematic beam tilt occurs during scanning, shifting the diffraction patterns on the detector. This shift was used to arrange the patterns into a two-dimensional array corresponding to the scanned region. The maximum tilt angle from edge to edge in the scan is ± 0.65°, which is sufficiently small to avoid interference with the measurements. Next, a center-of-mass algorithm was applied to adjust the beam shift for each diffraction pattern. Following indexing, 360 virtual detectors (0.04 × 0.04 Å^–1^) were positioned in a ring around the direct beam at a radial distance corresponding to the g-vector of the (200) reflections (Figure 1b). The intensity recorded within each detector was used to construct an intensity profile as a function of azimuthal angle. To enhance the signal-to-noise ratio, the angular ranges of 1–180° and 180–360° were combined to produce a final azimuthal intensity curve of 1–180°. Subsequently, a threshold value was calculated using the Fourier transform of the azimuthal curve by determining the ratio of the first frequency bin to the DC component, where a higher ratio indicates a more defined peak in the signal. This threshold value was used to filter out unreliable diffraction data. By analyzing the azimuthal curves extracted from the recorded diffraction patterns, a single direction, *q*’*_i_*, at each beam location was identified based on the position of maximum intensity.

Due to changes in the field of view caused by tilting the sample during data acquisition, the three data sets were scaled and aligned prior to reconstruction, utilizing the projective transform and warp functions from the Scikit-Image library.^36^ This ensured that each pixel represented diffraction information from the same spatial position in the sample.

After image alignment, the maximum-intensity directions for each pixel are used to calculate a direction vector *q*’_i_ = [cos *φ*, sin *φ*, 0]^*T*^, which falls on the circle orthogonal to the fibril axis. This direction is back-rotated to sample fixed coordinates at zero tilt by *q_i_* = R_y_(−*ω*_i_)*q*’_*i*_, where R_y_(*ω*) is the rotation matrix for a rotation about the *y*-axis by the tilt angle, *ω*.

The fibril axis for each pixel is then the unique direction that is simultaneously orthogonal to the vectors *q_i_* from each measurement in the tilt-series. Due to uncertainties arising from the measurement and image alignment, there will not be any unique direction; rather, a best-fit direction must be found. To solve this problem, an iterative algorithm called averaged projections was used, where projection onto the set orthogonal to *q*_*i*_ is given by

$$P_{i}\left(â\right)=â-\left|â\cdot q_{i}\right|q_{i}$$

For each iteration, the current estimate of the fibril axis â_*k*_ is projected onto each measurement in the tilt series, and the average of the projections is calculated:

$${\mathrm{b̂}}_{k}=\frac{1}{N}\overset{N}{\underset{i=1}{\sum }}P_{i}\left({â}_{k}\right)$$

Finally, this average is projected onto the unit sphere to give the estimate for the next iteration.

$${â}_{k+1}=\frac{{\mathrm{b̂}}_{k}}{\left|{\mathrm{b̂}}_{k}\right|}$$

## Supplementary Material





## Data Availability

SED data and Python script for SED data analysis used in this study are available from Zenodo (DOI: 10.5281/zenodo.15647651). Additional data are available from the corresponding author upon request.

## Notes

Effect of using positive and negative y-components to visualize helices of different handedness, out-of-plane angles in Figure 2 along with helix, circumference, and pitch equations, calculated helix angles and pitch values from the oat husk layers in Figure 2, the estimated orientational relationship between layers in Figures 3 and 4, mean out-of-plane angles in birch wood, the orientational relationship between birch wood layers, probe size evaluation, and the estimated error calculation (PDF)
An animation of Figure 2 (MP4)
