# Supplementary material for: Chiral Hierarchies at the Nanoscale Revealed by Three-Dimensional Scanning Electron Diffraction
Source: ACS Nano. 2025 Sep 30;19(40):35777–86. doi: 10.1021/acsnano.5c12291 (PMC12530053; doi:10.1021/acsnano.5c12291)
Supplement: Supplementary file 1 [file nn5c12291_si_001.pdf]

Supporting information

Chiral hierarchies at the nanoscale revealed by three-dimensional scanning electron diffraction

*Mathias Nero[1], Mads Carlsen[2], Marianne Liebi[2,3], Tom Willhammar[1]**

[1.] Department of Chemistry, Stockholm University, SE-106 91, Stockholm, Sweden

[*] E-mail: tom.willhammar@su.se

[2.] Photon Science Division, Paul Scherrer Institute, 5232 Villigen PSI, Switzerland

[3.] Institute of Materials, Ecole Polytechnique Fédérale de Lausanne (EPFL), 1015 Lausanne, Switzerland

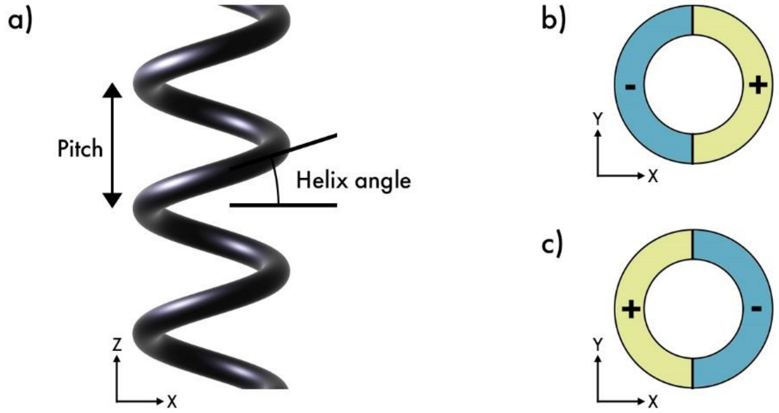

Figure S1. Effect of using positive and negative y-components to visualize helices of different handedness. A right-handed helix (a), viewed from the top (or bottom), exhibits a positive y-component on the right side of the center and a negative y-component on the left, resulting in the color pattern displayed in (b). In contrast, (c) presents a left-handed helix, leading to reversed y-components and, consequently, colors.

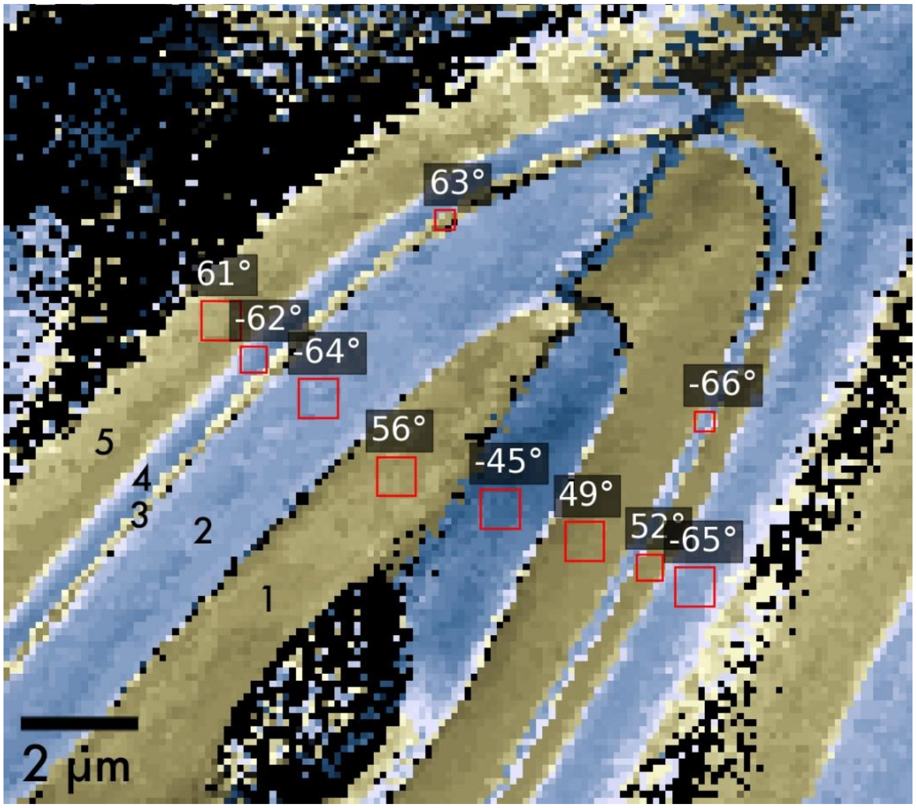

Figure S2. Out-of-plane color map of a transversely sectioned oat husk cell wall (from the dataset shown in Figure 2), with corresponding out-of-plane angles. Values on the right side of the lumen are generally lower than those on the left, likely due to slight deviation from ideal perpendicular sectioning. The estimated circumference (C) of the cell wall layers is derived from the STEM image in Figure 2b and is calculated:

$$C = \pi(3(a+b) - \sqrt{(3a+b)(a+3b)}$$

With:

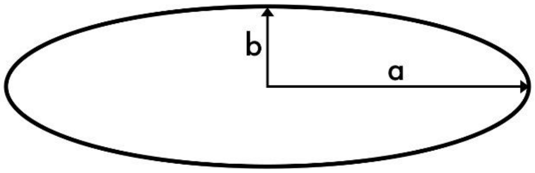

The helix angle (θ) is calculated as 90° minus the average of two out-of-plane measurements. For example, in layer 1:

$$\theta = 90° - \frac{|56°|+|-45°|}{2} = 39.5°$$

Pitch (P) is calculated:

$$P = \tan\theta \times C$$

Table S1. Calculated helix angles and pitch values from the oat husk layers in Figure 2 (and S2)

	Circumference	Helix angle	Pitch
Layer 1	31 μm	39.5°	25 μm
Layer 2	62 μm	33.5°	41 μm
Layer 3	72 μm	24.5°	32 μm
Layer 4	77μm	33.5°	50 μm
Layer 5	90 μm	27.5°	45 μm

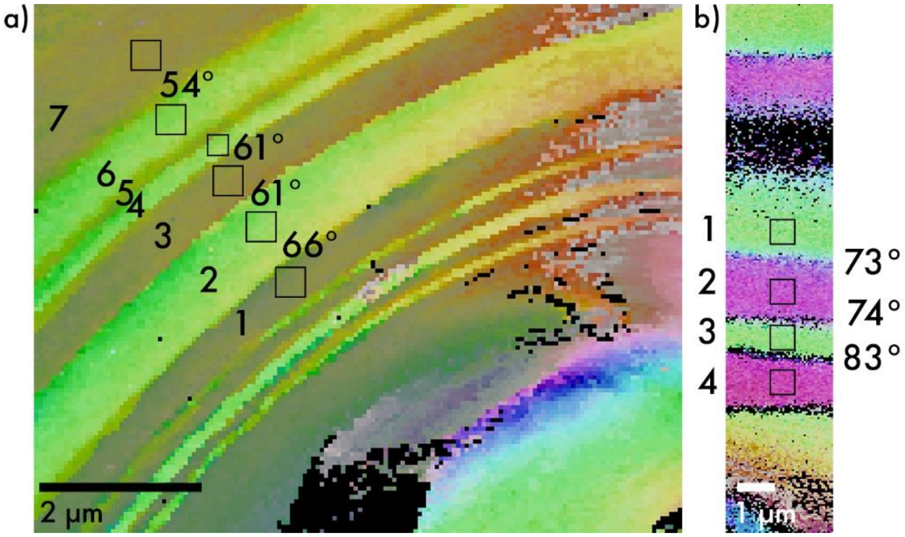

Figure S3. 3D-SED reconstruction color maps depict a transversely sectioned oat husk cell wall (Figure S3a) and a longitudinally sectioned oat husk cell wall (Figure S3b). The estimated orientational relationship between layers is determined by calculating the dot product of the two vectors. Since fiber orientation is non-directional, the difference between the two regions is defined as the smallest angle between their respective orientation vectors. The absolute value of the dot product between the averaged orientations of each layer, indicated by the black squares, quantifies the angular difference. For instance, the orientation relationship between regions a1 and a2 is 66°, while that between b1 and b2 is 83°.

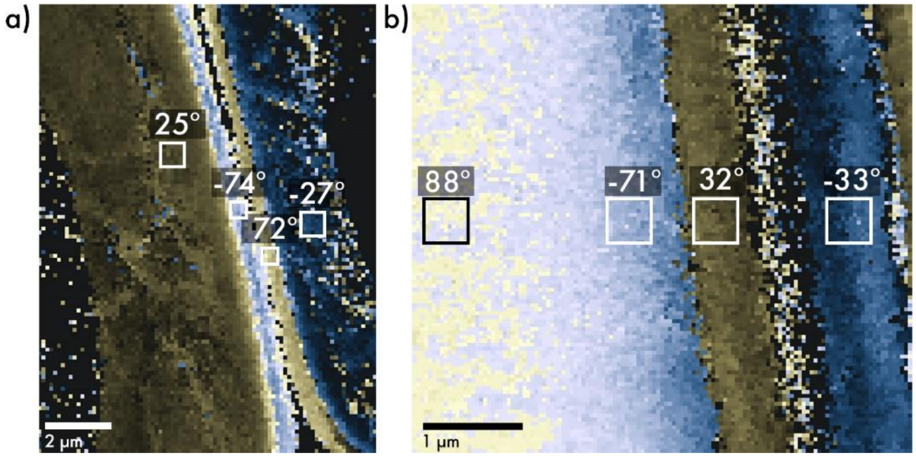

Figure S4. Out-of-plane visualizations of birch wood cells. (a) Transverse section and (b) longitudinal section, with mean out-of-plane angles indicated next to the squares in each image. An angle of 0° corresponds to fibril orientation normal to the sectioning plane, while 90° indicates alignment parallel to the plane.

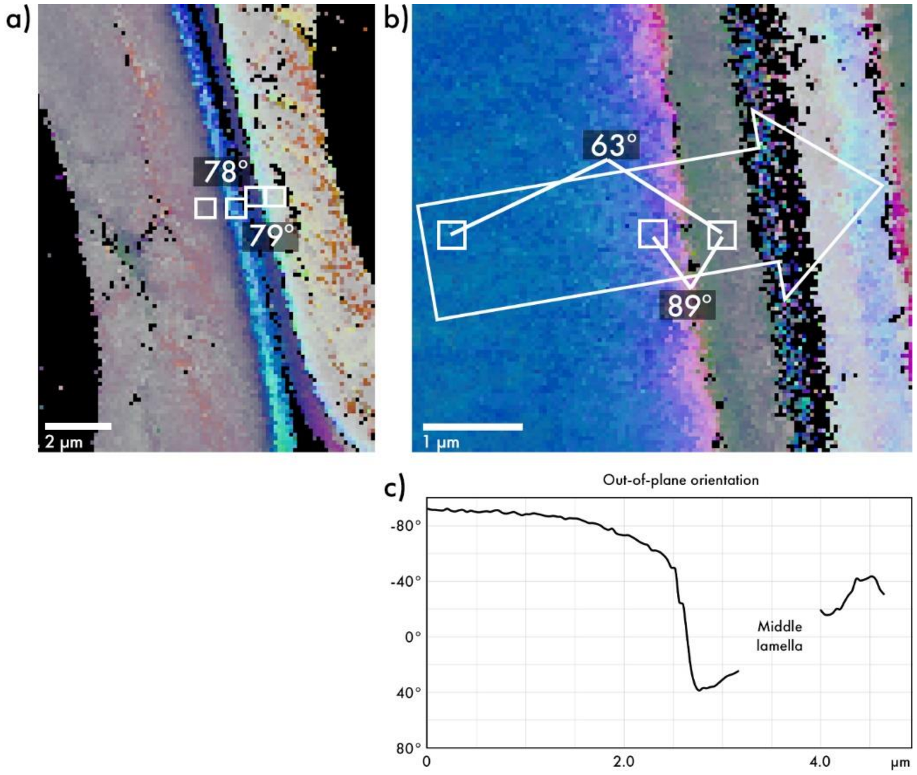

Figure S5. 3D-SED reconstruction color maps of birch wood cells in (a) transverse and (b) longitudinal sections. The orientational relationship between layers is calculated using the dot product. In (a), the angle between the two squares on the right is 78°, while the angle between the two squares on the left is 79°. In (b), the orientation relationship varies as fibril orientation changes: the absolute angle between two squares separated by 2.5 µm is 63°, whereas it is 89° between two squares that are closer together. The graph in (c), based on values extracted along the line profile indicated by the arrow, illustrates the smooth out-of-plane orientational transition from the thick inner layer to the thin outer layer, corresponding to a total rotation of 120°.

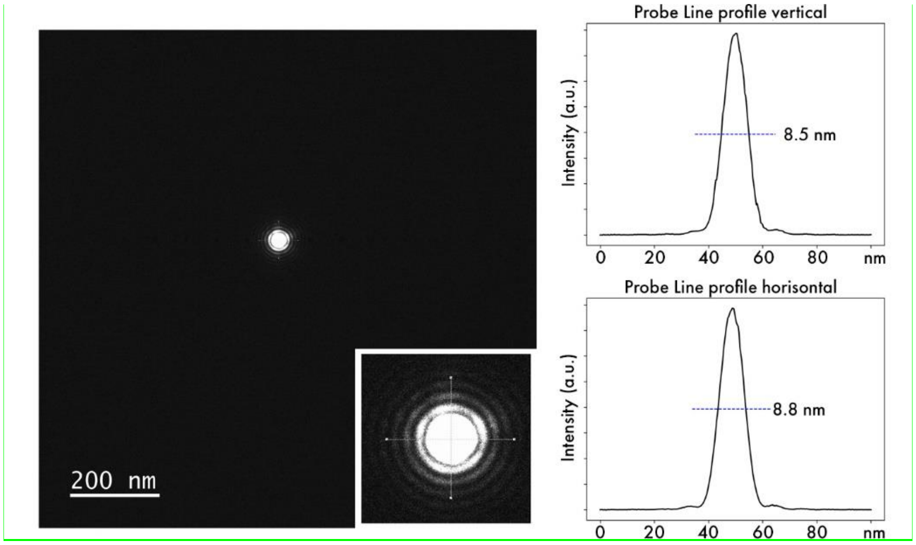

Figure S6. (a) TEM image of the probe with a magnified inset. (b) Vertical and horizontal line profiles of the probe indicate a probe diameter of approximately 8.7 nm at FWHM.

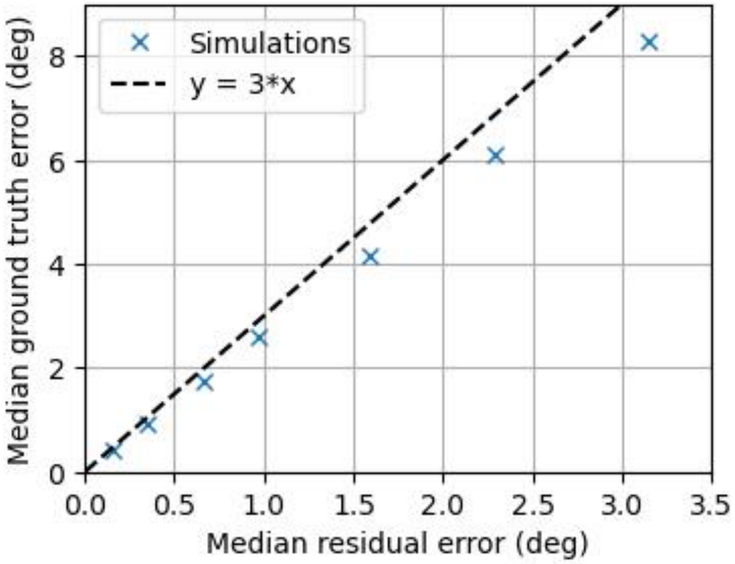

Figure S7. A set of synthetic datasets is generated by assuming a ground truth fiber direction a_i^*, and generating a set of measured azimuthal angles of directions orthogonal to these directions plus an added random noise term drawn from a normal distribution with a variance σ^2. From these synthetic datasets a best fit direction a_i is calculated and the accuracy the recovered direction is estimated by the mean angular error in degrees, $\text{Ground truth error} = \frac{1}{M}\sum_{j=1}^{M} \arccos(|a_j^* \cdot a_j|)$.

This estimated error is compared to the observed deviation of the measured direction from the set orthogonal to the recovered direction calculated as $\text{Residual Error} = \frac{1}{M}\sum_{j=1}^{M}\frac{1}{N}\sum_{i=1}^{N} \arcsin(|q_{ij} \cdot a_j|)$ where N is the number of points in the tilt series. S7 shows a linear correspondence between the mean observed residual error and the ground truth error with a slope of 3 for simulation using three images in the tilt series. The observed residual error within each layer of the experimental data is measured to be on the order of 1°, corresponding to an actual accuracy of about 3°.
